# Fine scale spatial variability of microbial pesticide degradation in soil: scales, controlling factors, and implications

**DOI:** 10.3389/fmicb.2014.00667

**Published:** 2014-12-05

**Authors:** Arnaud Dechesne, Nora Badawi, Jens Aamand, Barth F. Smets

**Affiliations:** ^1^Department of Environmental Engineering, Technical University of DenmarkLyngby, Denmark; ^2^Department of Geochemistry, Geological Survey of Denmark and GreenlandCopenhagen, Denmark

**Keywords:** biodegradation rate, leaching, hotspot, geostatistics, tillage, motility

## Abstract

Pesticide biodegradation is a soil microbial function of critical importance for modern agriculture and its environmental impact. While it was once assumed that this activity was homogeneously distributed at the field scale, mounting evidence indicates that this is rarely the case. Here, we critically examine the literature on spatial variability of pesticide biodegradation in agricultural soil. We discuss the motivations, methods, and main findings of the primary literature. We found significant diversity in the approaches used to describe and quantify spatial heterogeneity, which complicates inter-studies comparisons. However, it is clear that the presence and activity of pesticide degraders is often highly spatially variable with coefficients of variation often exceeding 50% and frequently displays non-random spatial patterns. A few controlling factors have tentatively been identified across pesticide classes: they include some soil characteristics (pH) and some agricultural management practices (pesticide application, tillage), while other potential controlling factors have more conflicting effects depending on the site or the pesticide. Evidence demonstrating the importance of spatial heterogeneity on the fate of pesticides in soil has been difficult to obtain but modeling and experimental systems that do not include soil's full complexity reveal that this heterogeneity must be considered to improve prediction of pesticide biodegradation rates or of leaching risks. Overall, studying the spatial heterogeneity of pesticide biodegradation is a relatively new field at the interface of agronomy, microbial ecology, and geosciences and a wealth of novel data is being collected from these different disciplinary perspectives. We make suggestions on possible avenues to take full advantage of these investigations for a better understanding and prediction of the fate of pesticides in soil.

## Introduction

Pesticide application onto agricultural fields is a common practice in modern societies. In Denmark alone, 5,715 T of pesticides were used in 2012 for agricultural purpose (Miljøstyrelsen, 2013). Many of these active compounds would be persistent in soil if degrading microbes were not present and active. In fact, if microbial biodegradation does not occur, or occurs insufficiently, these molecules may adversely affect the biosphere and contaminate our water resources (Arias-Estévez et al., 2008). As a consequence, pesticide degradation—whether partial transformation or complete mineralization to elemental substances–by soil microbes has received ongoing attention from microbiologists over the last 60 years. Historically, the emphasis has been on demonstrating the role of microbes in degradation, isolating the organisms involved, and determining the underlying degradation pathways and their genetic determinants. A large body of work has also been devoted to studying the influence of environmental parameters on the rate and extent of biodegradation. However, most of this research was done without explicitly considering fine scale spatial variability in degradation. Spatial variability was mostly considered at larger scale: landscape, catchment, or regional scale, where obvious heterogeneities in soil type, land usage, and hydrology made the assumption of spatial homogeneity difficult to defend. In this context, soil samples were often taken in a single location in a field or in a plot or, if several samples were collected, they were pooled and mixed in an attempt to provide a single “representative” sample.

Now, there is an increasing awareness that, even at a fine spatial resolution (within a field), pesticide biodegradation rates and potentials are not spatially homogeneous. A number of studies have been devoted to describing this spatial heterogeneity, identifying controlling environmental parameters, or—more rarely—exploring its consequence on overall degradation efficiency and risk of pesticide leaching. To our knowledge, there has been little effort to synthesize this primary literature and we consider a review of these findings timely. We also attempt to highlight current gaps in our understanding and propose avenues for future research.

## Why study the fine scale spatial heterogeneity of pesticide biodegradation?

The motivations to study the spatial heterogeneity of pesticide degradation at the field scale or at finer scales can be divided into two broad categories: applied and ecological. Specifically, some authors argue that such knowledge is important for pesticide management and risk assessment and that taking spatial heterogeneity into account would significantly improve models of pesticide fate in soil (Holden and Firestone, 1997; Soulas and Lagacherie, 2001). The other applied motivation is to guide sampling strategy and help make rational choices of sample volume and sample number to accurately describe field- or plot-wide biodegradation rates. Later, we will discuss if these claims of applied importance are supported by the literature.

There is mounting consensus that describing and understanding microbial spatial patterns in soil and their dynamics have significant ecological value. Spatial ecology has in fact been a fruitful sub-discipline of ecology for many decades, and the spatial ecology of soil microorganisms is receiving increasing attention (Ettema and Wardle, 2002; Dechesne et al., 2007; Nunan et al., 2007; Young et al., 2008). The spatial patterns of pesticide degraders can be considered as the integrated consequence of the ecological features of the degrading community (e.g., niche characteristics, dispersal ability) and of the extrinsic processes acting on the community (e.g., predation, pesticide fluxes). The spatial pattern of the degrading community can, in turn, affect the way the community is affected by ecological processes (e.g., gene transfer, predation). Again, this review will aim at verifying whether valuable ecological information has been extracted from spatially explicit approaches to study pesticide biodegradation or pesticide degraders. This review will focus on cultivated soils, which are major recipients of pesticides and for which most literature is available. These soils are subject to fluctuating conditions (e.g., changes in water and nutrient fluxes, modification in physical structure) associated with crop growth cycles, agricultural practices, and climatic events.

## Possible approaches to studying the spatial patterns of pesticide biodegradation–methodological observations

Table 1 and Table S1 list a number of papers that have described the spatial variability of pesticide degraders in agricultural fields. Before highlighting general trends in their findings, one has to acknowledge the differences in approaches and methodologies. First, while a few studies focused on *in situ* degradation, most report measurements made on samples brought back to the laboratory (Table S1). The former approach attempts to monitor degradation as it takes place in soil, while the latter, by exposing the samples to controlled (and often “optimal”) conditions, gives access to the microbial degradation potential present in the sample, to the expense of some of the relevance to field conditions. Laboratory assessment of biodegradation often relies on monitoring ^14^CO_2_ evolved from soil microcosms spiked with a ^14^C–labeled pesticide. These studies estimate the mineralization potential of the sample, which is more restrictive than the potential for biodegradation or biotransformation of the parent compound. Second, very different metrics are reported for quantifying biodegradation from biodegradation curves: e.g., maximum extent of biodegradation, maximum biodegradation rate, lag phase before onset of biodegradation, time to a certain percentage of compound degradation.

**Table 1:** **Summary of the main findings (description of variability and co-varying parameters) presented in the primary literature on within-field spatial variability of microbial pesticide degradation potential**.

**Pesticide (previous exposure)**	**Extent of the sampled area^‡^**	**Spatial variability of degradation/mineralization potential (coefficient of variability, when available)**	**Association with other parameters^†^**	**Reference**
2,4-D (no)	50 cm^2^; 1 and 50 m^2^	High at mm scale (CV up to > 150%), lower at m scale (< 22%)	ND	Vieublé Gonod et al., 2006
2,4-D (no)	26 cm^2^	Highest for smallest soil aggregates (CV 67% for the 2–3.15 mm class)	ND	Gonod et al., 2003
2,4-D (no)	79 cm^2^; 36 mm^2^	High in dry condition (CV 65%), low in wet conditions (14%),	+ with *tdfA* (only after incubation with 2,4-D; **0** with 16S rRNA)	Monard et al., 2012
2,4-D (yes)	360 m transect	Highly variable in each horizon	in A horizon: **–** with OM, pH, carbonate content, and 2,4-D sorption; **0** in the other horizons	Gaultier and Farenhorst, 2007
2,4-D (yes)	300 m transect	High variability across small distances	**0** with SOC and clay content	Shymko and Farenhorst, 2008
MCPA (yes)	3 10^4^ m^2^	Low in A horizon (CV 5%), high in B (56%)	**0** with Clay, C_org_, pH, ISR, CFU, *Pseudomonas*, SIR, ASA, FDA, *tfdA*	Fredslund et al., 2008
MCPA (no, but old exposure to DCPP)	68 cm^2^	Variability increases with depth, until no degradation is detected (115 cm)	ND	Badawi et al., 2013b
Linuron (yes), MCPA (no)	400 m^2^	Low for MCPA; high for linuron	+ with pH and water- extractable PO^3+^_4_; **0** with CFU (heterotrophs); **–** with C_total_/N_total_ ratio	Rasmussen et al., 2005
Isoproturon (yes)	3600 m^2^	CV 32%	+ with pH (mainly) and microbial C; **0** with community fingerprint and physico-chemical parameters	El Sebai et al., 2007
Isoproturon (yes)	Two 100 m transects, separated by 50 m	High	ND	Bending et al., 2001
Isoproturon (yes)	1120 and 1 m^2^	Higher at field scale (41%) than within 1 m^2^ (18%)	ND	Beck et al., 1996
Isoproturon (yes)	1.3 10^4^ m^2^	Low after IPU application (16%), increases subsequent years (54 and 45%)	+ with CFU, pH (weak), C/N; **0** with bacterial diversity; **–** with total N, CEC, humidity	Hussain et al., 2013
Isoproturon (yes)	5.8 10^4^ m^2^	Overall low (35%), but over 50% of the within-field variability occurs < 27 m	+ for pH; microbial biomass; **–** with SOM (weak); **0** for adsorption coefficient	Price et al., 2009
Isoproturon (yes)	5 10^4^ m^2^	Significant within-field spatial variability, relatively high temporal consistency	++ with pH and biomass; + with metabolic richness and diversity	Walker et al., 2001
Isoproturon (yes) and chlorotoluron (no)	1260 m^2^	Strong variability at scales < few m	+ with pH; positive covariation of degradation rate of the 2 pesticides.	Walker et al., 2002
Isoproturon (yes), azoxystrobin (yes), diflufenica (yes)	9600 m^2^	CV between 14 and 56%	+ with pH for azoxystrobin; **0** with C/N, microbial N, dehydrogenase activity	Bending et al., 2006
Isoproturon (yes), bentazon (no), mecoprop (no)	1.4 10^4^ m^2^	Higher for subsoil than topsoil (e.g., for bentazone 44% vs. 16%)	+ with OM, DHA (all pesticides); **–** with pH (bentazon). In subsoil for isoproturon and mecoprop: + with sand content; – with clay and silt contents	Rodriguez-Cruz et al., 2006
Bentazone (no)	3 plots separated by 60 m	Higher in topsoil (15–30%) than in subsoil (~ 7%)	+ with biomass, DHA, OM, and moisture content for sieved soil; **–** with pH	Rodriguez Cruz et al., 2008
Glyphosate (no), metribuzin (no), and riazinamin (no)	4.0 10^4^ m^2^	Higher at field scale (61%) than at local scale (25%) for glyphosate (others not degraded)	+ with clay, C_org_, ISR, SIR, ASA, FDA; **0** with CFU and *Pseudomonas*	Vinther et al., 2008
Glyphosate (?)	3240 m^2^; 1 m transect	CV ~ 30%	+ (but weak) with C_org_, and moisture content	Stenrød et al., 2006
Simazine and metribuzin (?)	6400; 1600; and 9 m^2^	Higher for metribuzin (~ 23%) than for simazine; affected by mode of pesticide application	ND	Walker and Brown, 1983
Carbofuran (yes)	Transects > 50 m in adjacent row and inter-row	Higher in no till field (~ 33%) than in conventional tilled filed (~ 15%)	+ with moisture content	Parkin and Shelton, 1992
BAM (parent compound)	50; 1; and 0.01 m^2^	Increases from 60% to 108% with decreasing spatial scale and sample volume	+ with MPN of BAM degraders; **0** with TOC, pH, moisture content, NH^+^_4_, and NO^−^_3_	Sjoholm et al., 2010

*See Table S1 for more information on the sampling design of these studies*.

‡ *Multi-scale sampling schemes: values separated by semi-colon*.

† *Positive, negative, and absence of significant association (or correlation) are indicated by +, −, and ***0***, respectively; ND: not determined; OM: organic matter; SOC: soluble organic carbon, C_org_: organic carbon; ISR: In situ soil respiration; SIR: substrate induced respiration; ASA: arylsulfatase activity; FDA: fluorescein diacetate hydrolysis activity; DHA: dehydrogenase activity; CEC: cation exchange capacity; CFU: colony forming unit; MPN: most probable number*.

Another obvious difference between studies listed in Table S1 lies in the sampling strategy: the dimension of the sampled area, the distance between samples, sample mass, whether or not samples are pooled together, and whether or not the soil structure is disrupted. All these factors vary largely from study to study; for example, the distance between neighboring samples varies by more than 4 orders of magnitude (Table 2). The sampling strategy in some of the papers is poorly explained. This obviously makes it difficult to rigorously compare results across studies.

**Table 2:** **Horizontal scales of variation considered in the primary literature**.

	**Horizontal distance between neighboring samples (m)**				
	**≤ 0.1**	**0.1–1**	**1–10**	**10–100**	**100–250**
Percentage of references	29	21	29	67	13

We categorized the horizontal distance between neighboring samples in the references listed in Table S1 in 5 bins. The total is more than 100% because many references (38%) have multi-scale sampling schemes.

A last notable element is the way data are analyzed. On the one hand, some studies do not completely take advantage of their spatially explicit sampling scheme. This is the case when variability is only described in terms of coefficient of variation (CV) across sampling locations (13% of the references in Table S1), or when correlation or multiple regression are used to identify environmental parameters that co-vary with degradation rate across sampling points (46% of the references). On the other hand, some authors use spatial statistics such as geostatistics to describe the spatial variability of pesticide degradation or degradation potential, and to test whether non-random spatial structures are present and can be related to environmental variables (33% of the references). In geostatistics, the spatial structure is truly seen as pertinent information in its own right. Applying geostatistics requires a relatively large number of samples, possibly justifying why many authors have limited their analysis to non-spatial descriptive statistics.

## Scale and extent of the spatial variability of biodegradation potential

Researchers have explored spatial variability both horizontally and vertically (Table 1 and Table S1). These studies clearly indicate that the spatial pattern of degraders is not isotropic, as variation with depth differs from horizontal variation.

### Variation with depth

Consistent decline in average pesticide biodegradation rate with increasing soil depth has been reported for diverse compounds (Larsen et al., 2000; Rodriguez-Cruz et al., 2006; Stenrød et al., 2006; Gaultier and Farenhorst, 2007; Fredslund et al., 2008; Rodriguez Cruz et al., 2008; Shymko and Farenhorst, 2008; Badawi et al., 2013b). This reduction in mineralization potential is detectable over relatively short vertical distances from the plow layer and into the subsoil, but sometimes extends down to the underlying aquifer (Batıoğlu-Pazarbaþı et al., 2012), and follows a general trend similar to that of total bacterial biomass (Holden and Fierer, 2005). This decrease can be related, with some confidence, to a gradient of decreasing resource flux, as the most significant input of organic substrates (including pesticides) takes place in the uppermost part of the soil profile. It has however been observed that the biodegradation potential can sometimes decrease more steeply than the total heterotrophic population (Rodriguez Cruz et al., 2008; Badawi et al., 2013b). This was the case for MCPA degraders in a soil with no history of MCPA application, where a 100-fold decrease in biodegradation potential was observed between the plow layer and 115 cm depth, compared to 10-fold for the total heterotrophs (Badawi et al., 2013b). This relative enrichment of degraders in top layers could be linked to the fact that microbial communities at the surface are more exposed to fresh input of substrate compared to their subsoil counterparts. Pesticide-degrading potential can extend to the groundwater table and further down into the underlying aquifer sediments below soils repeatedly treated with pesticide (Batıoğlu-Pazarbaþı et al., 2012), highlighting how a history of pesticide application can modulate the vertical variability of pesticide biodegradation.

The vertical decline of mineralization potential is often associated with an extension of the lag phase prior to rapid biodegradation in degradation batch assays, rather than by a decrease in actual biodegradation rate (Bending and Rodriguez-Cruz, 2007; Badawi et al., 2013b). This is consistent with a control of vertical variability by initial degrader abundance, as the lag phase likely corresponds to the time necessary for the initial degrading population to reach a significant density (Rosenbom et al., 2014). However, since this lag phase can be several weeks long, it is possible that, under field conditions, mobile pesticides could be transported downwards before much degradation happens.

### Horizontal variability

Horizontal patterns of degradation potential cannot be related to environmental gradients as obvious as depth gradients. In this plane, extracting common trends proves much more challenging as the horizontal distribution of pesticide degraders appears to vary widely depending on the pesticide, the sampling design, and possibly the site considered.

In surface soil, pesticide degradation potential can be characterized by low coefficients of variation, either because the pesticide is homogeneously poorly degraded [e.g., metribuzin and triazinamin in Vinther et al. (2008)] or because it is readily degraded in most samples [e.g., MCPA in top soil samples collected both at the field scale and across a few cm^2^ (Fredslund et al., 2008; Badawi et al., 2013b)]. High CVs, often exceeding 40%, are associated with pesticides that show contrasting degradation potential across the sampled area [e.g., glyphosate (Vinther et al., 2008), linuron (Rasmussen et al., 2005), and isoproturon (Rodriguez-Cruz et al., 2006)]. Compared to variation in physical soil properties, these variability values are rather high (Mulla and McBratney, 1991), as it is the case for many other types of microbial activities (Amador et al., 2000).

At scales smaller than the field scale, the horizontal variability for pesticide degradation potential tends to increase as the studied area is smaller (Walker and Brown, 1983; Vieublé Gonod et al., 2006; Sjoholm et al., 2010). This is consistent with the existence of a high spatial variability in biodegradation at small scale, which is possibly even magnified by the fact that smaller sample volumes typically used to measure small scale variability are more sensitive to the effect of small spots of high- or low activity compared to larger sample volumes. Indeed, 2,4-D biodegradation in 2–3 mm aggregates presented higher variability (CV = 67%) than in slightly larger aggregates (CV about 40% for 3–5 and 5–7 mm aggregates) (Gonod et al., 2003). A few authors, however, identified an opposite trend with higher CVs at the field scale than at the meter to tens of meters scale (Beck et al., 1996; Vinther et al., 2008). This would be consistent with spatially auto-correlated degradation potential with ranges extending up to several meters (see Table 3 for a definition of these terms). CV as high as those reported at the field scale (~50%) have been measured for the degradation rate of various pesticides at the scale of a small catchment (Ghafoor et al., 2011a), even though the 13 km^2^ catchment comprised several classes of soil, and thus significant heterogeneity in environmental conditions.

**Table 3:** **Brief definition of terms used in spatial statistics and geostatistics [see for example (Mulla and McBratney, 1991) for more detailed information]**.

**Term**	**Definition**
Spatial autocorrelation	Property of a spatially structured variable such that observations collected close to each other are more similar (or less similar, for negative autocorrelation) than observations from more distant samples
Range	In geostatistics, this is the distance within which observations show significant autocorrelation and beyond which observations can be deemed truly independent
Nugget effect	In geostatistics, this indicates the existence of variability at scales smaller than the minimum inter-sample distance. This variability can originate from measurement or sampling error, or from the existence of small-scale spatial variability in the measured variable

**Figure 1:**
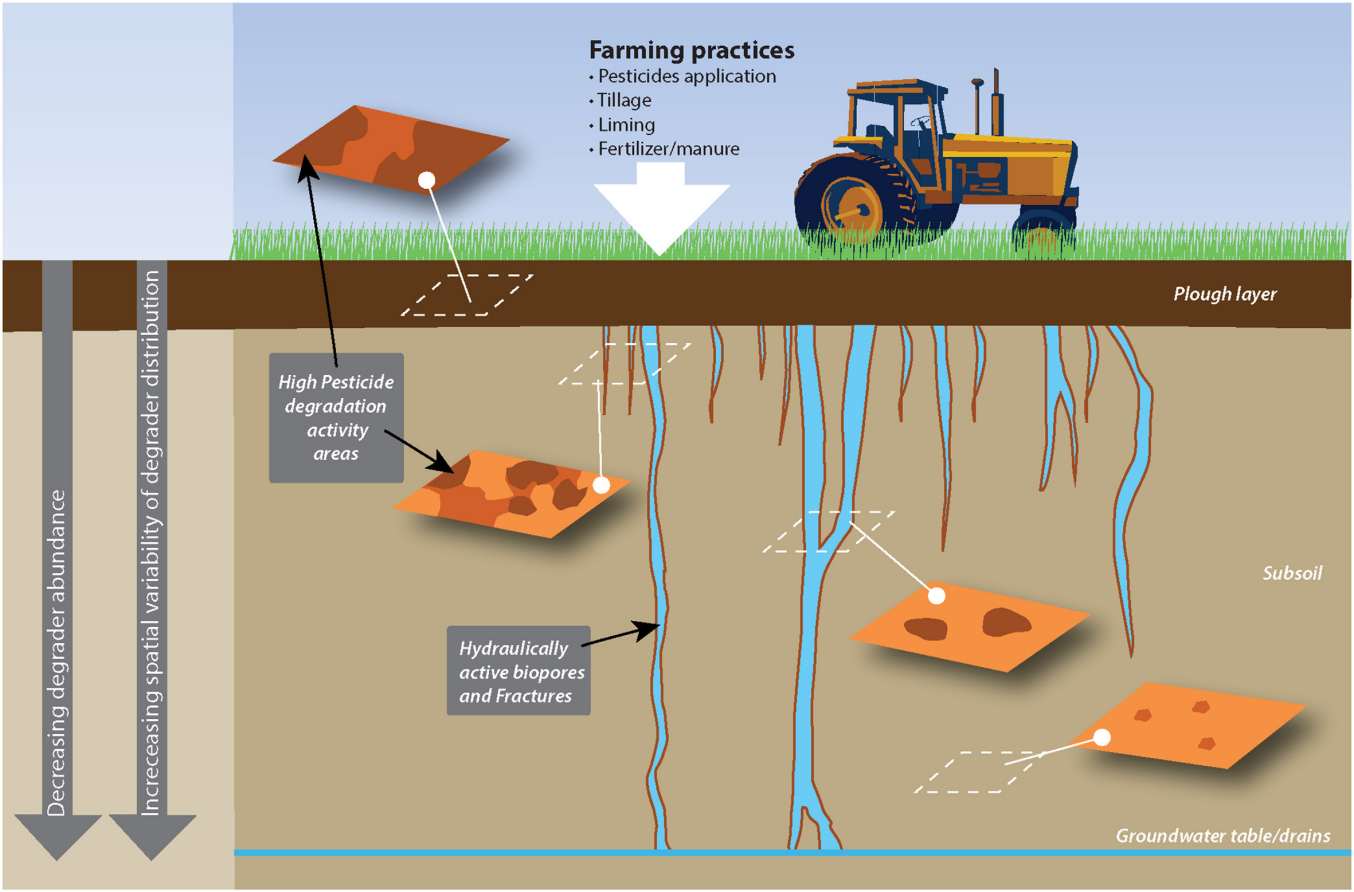
**Example of the horizontal and vertical spatial distribution of pesticide degradation as affected by agricultural practices and soil structure**. This sketch presents the distribution of MCPA degraders in a soil treated with MCPA and where MCPA is mainly transported through subsoil via wormholes, as observed by rensen and Aamand (Badawi et al., 2013a,b). Surface soil, which receives MCPA application, hosts a relatively abundant and spatially homogeneous degrading population, while degraders in the subsoil cluster around preferential flow paths.

Importantly, horizontal variability is also found to vary with depth, with a tendency for larger variability in deeper soil layers (Rodriguez-Cruz et al., 2006; Gaultier and Farenhorst, 2007; Fredslund et al., 2008; Vinther et al., 2008; Badawi et al., 2013b). In a recent study describing the centimeter scale distribution of MCPA degraders (Badawi et al., 2013b), degraders were found to be homogeneously distributed in surface soil, while, below the plow layer, they formed a few hotspots separated by zones of soil devoid of degradative potential (Figure 1).

In general, the detection of non-random spatial pattern depends on the scale considered. For example, Vieublé-Gonod and colleagues failed to identify significant spatial pattern of 2,4-D biodegradation potential at scales larger than a few centimeters, while spatial dependency in biodegradation rate was apparent for distances smaller than 2 cm (Vieublé Gonod et al., 2006). In cases where geostatistics have been used in combination with a solid sampling effort, significant spatial structure (spatial auto-correlation) is commonly detected at least at one scale (Table S1). For example, ranges (see Table 3 for definition) up to 40–50 m are reported for isoproturon (El Sebai et al., 2007) and carbofuran (Parkin and Shelton, 1992). The presence of structural dependencies at scales smaller than the minimum sampling distance is often suggested by the occurrence of significant nugget variance (Table 3), although nugget variance also includes variance originating from measurement errors (Parkin and Shelton, 1992). Overall, it appears that the existence of several distinct scales of spatial dependence (i.e., nested spatial structure) is common for pesticide biodegradation potential in agricultural soils.

## Controlling factors and co-variants

### Methodological aspects

Many field studies are of descriptive nature and attempt to detect parameters that significantly co-vary with biodegradation rate, rather than to formally identify controlling factors. These studies can obviously serve as the base for hypothesis formulation, and subsequent hypothesis testing. Often, several of the measured biological and physico-chemical parameters are cross-correlated, making the identification of “true” controlling factor(s) elusive, and—often—the true controlling factor might not have been measured. This limitation is for example obvious for biodegradation variability across soil depth as many of the commonly measured soil parameters vary with depth and their correlation with pesticide biodegradation is likely to be spurious (i.e., devoid of causal basis).

Another methodological aspect might limit our ability to identify controlling factors of biodegradation potential heterogeneity. If one can argue that the association between microbial activity and their environment should be looked for at a fine scale [i.e., close to the micro-habitat scale, the scale at which microbes experience their environment (Parkin, 1993; Nunan et al., 2007; Vos et al., 2013)], our current experimental techniques are not sophisticated enough to measure biodegradation rates and multiple other parameters in samples weighing only a fraction of a gram. Correlation approaches are thus challenging or even impossible at the scale where they could be the most informative (Gonod et al., 2006).

Finally, there are statistical limitations associated with simple correlation approaches for spatially explicit data sets. Indeed, most standard tests rely on the assumption of independently and identically distributed errors, which is typically violated for spatially structured variables. In extreme cases, this may lead to an inversion of the conclusion of an analysis (Kühn, 2007). Fortunately, there exist ways to deal appropriately with such auto-correlated data sets for example using geostatistical approaches (Dormann et al., 2007), but they are rarely used in the literature we reviewed.

### Physico-chemical parameters

Many soil physico-chemical parameters have been measured in conjunction with biodegradation rates in order to look for covariates (Table 1). Unfortunately, there have been few significant covariates consistently identified across pesticides and sites. One partial exception to this is pH, which has been shown to be a good predictor of the biodegradation rate of many pesticides, such as bentazone (Rodriguez-Cruz et al., 2006), the phenyl-urea herbicides isoproturon (Bending et al., 2001, 2006; Rodriguez-Cruz et al., 2006; El Sebai et al., 2007; Hussain et al., 2013) and linuron (Rasmussen et al., 2005), and the fungicide azoxystrobin (Bending et al., 2006). pH is known to have major selective effects on soil microbiota (Lauber et al., 2009) and can modulate the sorption of many pesticides (Franco et al., 2009). Accordingly, the influence of pH on pesticide biodegradation has for example been noted at the global scale for the herbicide atrazine across soils differing by as much as three pH units (Houot et al., 2000). Within-field studies identified correlation between pesticide degradation and pH with pH variations within one pH unit or less (Bending et al., 2001, 2006; Walker et al., 2001; Rasmussen et al., 2005; Rodriguez-Cruz et al., 2006).

Soil moisture is another soil characteristic found to notably influence the horizontal variability of biodegradation across several classes of pesticide (Parkin and Shelton, 1992; Stenrød et al., 2006; Rodriguez Cruz et al., 2008; Monard et al., 2012; Hussain et al., 2013). In fact, the role of soil humidity is likely to be even more prevalent than currently appreciated because it is often not evaluated, for example when biodegradation is measured in soil slurries. Similarly to pH, water has fundamental effects on basic processes associated with microbial life. In addition to its physiological importance, soil water is also essential to solute movement in soil (Or et al., 2007).

Other parameters have less frequently been identified as covariates of pesticide biodegradation potential. These include C/N ratio and potassium content for linuron (Rasmussen et al., 2005), and organic matter and clay for glyphosate, bentazone, and mecoprop (Rodriguez-Cruz et al., 2006; Vinther et al., 2008). The contribution of the two latter parameters probably stems from their role in pesticide sorption.

Collectively, some of these physico- chemical characteristics can account for a large part of the spatial variability in biodegradation potential. This is especially true for phenyl-urea pesticides: 86% of the spatial variability of linuron biodegradation potential could be explained by a model including the observed values of pH, C/N, and phosphate content (Rasmussen et al., 2005) and, for isoproturon, up to 84% could be explained by pH and humidity (Hussain et al., 2013).

### Biological parameters

The typically evaluated biological parameters range from very general (e.g., biomass content, heterotrophic activity) to more specific (e.g., abundance of specific microbial groups, enzymes, or even degradative genes). The most general parameters typically do not present significant association with biodegradation rate. For example, the density of culturable cells did not correlate with biodegradation rate for the relatively recalcitrant pesticides glyphosate (Vinther et al., 2008) and linuron (Rasmussen et al., 2005), nor for the more easily degradable phenoxy acid herbicide MCPA (Fredslund et al., 2008). The same was true for total bacterial abundance measured by qPCR on the 16S rDNA (Monard et al., 2012). This is probably because pesticide degraders make up for minor fractions of the total microbial community and are thus not reliably assessed by community level methods. Similarly, the total community diversity is a very poor predictor of biodegradation rate (El Sebai et al., 2007), presumably because degraders are not among the dominant groups, which contribute most to diversity fingerprints. Vinther et al. (2008) did however identify significant association between glyphosate biodegradation and activity indicators such as substrate induced respiration and arylsulfatase activity.

Molecular based approaches have been used to assess specific communities that have a putative role in biodegradation. While these targeted approaches have sometime been successful, for example for the *Aminobacter* group in a BAM-contaminated soil (Sjoholm et al., 2010), they did not yield significant correlation for Pseudomonads and biodegradation of glyphosate (Vinther et al., 2008) or MCPA (Fredslund et al., 2008). This is presumably because the active degraders did not belong to this group or because Pseudomonads constitute a too large and too diverse group with only a minority of degraders. This last reason likely explains why the abundance of the common soil genus *Sphingomonas* is a poor predictor of isoproturon biodegradation (Shi and Bending, 2007).

Focusing on narrow groups of degraders or degradative genes is also often unsuccessful because initial degrader populations are typically below the detection limit of both culture-based and molecular quantification methods. In fact, these methods can usually only detect the target populations after they have proliferated following pesticide spiking, which obviously limits their predictive use. This was for example the case for qPCR-based detection of *tfdA* genes, associated with phenoxy acid degradation (Bælum et al., 2012; Monard et al., 2012), for qPCR based quantification of *Sphingomonas* phylotypes responsible for rapid isoproturon degradation (Bending and Lincoln, 2003; Shi and Bending, 2007), and for culture-based (most probable number) quantification of isoproturon degraders (Bending and Rodriguez-Cruz, 2007).

### Soil structural discontinuities

Cultivated soils present some obvious structural discontinuities (e.g., macropores, fractures, soil horizons) originating from agricultural practices, or from the intrinsic growth and activity of soil biota. It is tempting to relate these to the spatial heterogeneity of pesticide biodegradation.

#### Macropores and fissures

As mentioned above, pesticide biodegradation in subsoil is often very heterogeneously distributed, forming few discrete centimetric hotspots (e.g., Badawi et al., 2013b for MCPA). Vertical macropores, such as those created by earthworms, are known to be the preferential conduits for transport of soluble substrate from topsoil to subsoil (Nielsen et al., 2010). This preferential transport mechanism has been evidenced for pesticides such as atrazine (Edwards et al., 1993). It is thus plausible that biodegradation hotspots in subsoil occur close to these macropores. This hypothesis is largely supported by the literature for phenoxy acid pesticides (Pivetz and Steenhuis, 1995; Mallawatantri et al., 1996), where higher mineralization was reported in the macropore linings than in the surrounding soil matrix. This enhanced biodegradation is often underpinned by higher abundance of specific degraders [e.g., for phenoxy acid herbicides (Liu et al., 2011; Badawi et al., 2013a), atrazine (Monard et al., 2008)], but sometimes just by a general increase in growth activity, without enrichment of degraders [e.g., bromoxynil (Badawi et al., 2013a)]. The enrichment of degraders at the pore wall linings could be a consequence of beneficial conditions such as significant oxygen flux or availability of earthworm-derived growth substrates. Even if it is difficult to compare studies that often differ in their macropores selection (e.g., macropores presenting evidence of current or recent earthworm activity vs. merely hydraulically active macropores), macropores seem to importantly influence the spatial distribution of pesticide biodegradation hot-spots in subsoil.

#### Rows and furrows

Vegetal cover and agricultural management also introduce significant within-field spatial heterogeneities that can affect belowground microbial communities. Not surprisingly, these effects sometime extend to pesticide degraders and their activity (Alletto et al., 2010). For example, conventional tillage with a mouldboard plow was found to create more spatial heterogeneity in diketonitrile mineralization than conservation tillage (Alletto et al., 2008). This spatial variability was related to vertical and lateral variations in soil structure and organic matter distribution generated by these two types of tillage. A stimulatory role of increased C content and changes in the physical structure of the soil due to agricultural practices was also identified for isoproturon biodegradation. The presence of compost in inter-furrows enhanced biodegradation by about 10% and for up to 8 months after application relative to controls without compost amendment (Vieublé-Gonod et al., 2009).

### Pesticide application

Many pesticides can be metabolized by bacteria, which thus derive energy, carbon, and sometimes other elements (N, P) from their degradation. Consequently, pesticide application can stimulate the growth of the degrading populations, provided that they do not degrade it co-metabolically. Because pesticide application is typically discrete both in time and space, it can contribute to the spatial and temporal heterogeneities of the abundance of degraders and affect their spatial pattern. Evidence of this has been provided as early as 1983, when simazine biodegradation potential was shown to be more spatially variable in plots sprayed with a boom sprayer—resulting in more heterogeneous pesticide deposition–than in those treated using a knapsack sprayer- resulting in more homogeneous pesticide deposition (Walker and Brown, 1983). A similar localized enrichment of carbofuran degraders in maize rows, where the pesticide was applied, has also been reported (Parkin and Shelton, 1992). Pesticide application can also promote a homogenization of the spatial pattern of degraders. For example, isoproturon treatment on a wheat field enhanced the average biodegradation potential and homogenized the distribution of this potential, probably because the pesticide application was spatially homogeneous at the relatively coarse scale considered in this study (about 20 m separation distance between samples) (Hussain et al., 2013). In absence of further isoproturon amendment, the mineralization potential then declined for the next subsequent years and presented increasing field-scale variability. The homogenizing effect of repeated pesticide application has also been demonstrated for 2,4-D in repacked soil columns: before 2,4-D treatment, mineralization potential was limited to only a few patches, while repeated 2,4-D application resulted in the growth and spatial dispersal of the degrading populations (Pallud et al., 2004). Pesticide application is thus an important driver of the spatial distribution of pesticide-metabolizing communities. It can either result in increased or decreased spatial variability of pesticide biodegradation depending on the scale at which degradation is assayed compared to that of the variability of pesticide treatment.

## Consequences of spatial heterogeneity

### Sampling strategy and hypothesis testing

To the best of our knowledge, there is little example in the literature where quantification of the spatial distribution of biodegradation potential has been used to inform further sampling strategy or to adapt hypothesis testing. This is unfortunate because optimal sampling strategies should integrate information on spatial variability (Parkin, 1993). In particular, the presence of spatial autocorrelation diminishes the amount of information per sample, which thus should be compensated for to rigorously estimate and compare mean degradation rates (Mulla and McBratney, 1991). Precise guidelines have been lain out by Smith and coworkers (Smith et al., 1987) to adapt sampling effort to reach a target precision for spatially variable parameters in the context of pesticide fate evaluation, but these appear to have not been much adopted. It is likely that describing spatial heterogeneity is often considered experimentally too labor-intensive to serve as what can be perceived as “preliminary experiments.”

### Ecology of degraders

Overall, studies of the spatial distribution of pesticide degraders have so far contributed only modestly to improving our knowledge of their ecology. One of the biggest achievements may have been the demonstration that rapid phenylurea pesticide degradation in several agricultural soils is associated to *Sphingomonas* populations that have a narrow pH optimum (Bending and Lincoln, 2003; Shi and Bending, 2007). Other ecological processes, such as horizontal gene transfer, require cell-to-cell contact. In this context, the spatial distribution of indigenous 2,4-D degraders was taken into account to estimate their probability of contact with a bacterial population introduced to soil (Dechesne et al., 2005).

### Fate of pesticide

It is critical to establish whether, and under which conditions, the spatial distribution of degradation potential impacts pesticide fate, in term of persistence in soil or of risk of leaching. These are rather complex questions to address because, in natural systems, the spatial distribution of degraders is not known a priori and cannot be experimentally controlled in order to make replicated, comparative experiments. Therefore, researchers have resorted to either conducting experiments in simplified laboratory systems, or to using mathematical or computational models.

In repacked soil columns, it was shown that 2,4-D degradation was highest when the degraders formed the most disperse pattern (Pallud et al., 2004), which the authors ascribed to a better interception by the degraders of the 2,4-D flux percolating through the column. Additional direct evidence of the importance of the spatial distribution of degraders has not been established for pesticide degradation but for other organic compounds. For example, Dechesne et al. (2010a) manipulated the distribution of benzoate degraders in sand microcosms and measured significant differences in microcosms-wide benzoate degradation rates depending on the number of degradation hotspots introduced. In microcosms inoculated with 9 separated hotspots benzoate was degraded about 4 times as fast as in those containing only a single central hotspot, even though the initial total abundance of degraders was identical. With the help of a spatially explicit model, this observation was attributed to differences in diffusion limitation (Dechesne et al., 2010a). In a similar fashion, models developed by Banitz and collaborators confirmed, in soil-free systems, that a limited spatial occupancy of degraders results in diffusion-limited degradation, a situation that can be improved by degrader dispersal (Banitz et al., 2011a,b).

Spatial variability has increasingly been recognized as a potential source of uncertainty in standard pesticide fate models (Soulas and Lagacherie, 2001; Dubus et al., 2003). These models [e.g., MACRO, PEARL, and PRZM, (Beulke et al., 2001; Bouraoui, 2007; Farenhorst et al., 2009)] are typically one-dimensional and incorporate a single value of dissipation factor (DT_50_, time required for dissipation of half the initial pesticide concentration) as a measure of the pesticide degradation rate. This value is obtained from batch experiments with homogenized topsoil. If MACRO and PEARL are designed to be able to accommodate two domains, i.e., both a macro- and micro-porous one (Larsbo et al., 2005), this capability is rarely used because the relevant dissipation parameters are generally not available.

Beyond this dual-domain approach, the availability of affordable computing power makes it possible to develop models that include richer description of the spatial variability of pesticide degradation. At this point, examples in the literature are mostly limited to region- or catchment- scale variability. For example, simulation of atrazine leaching was strongly affected by the inclusion of spatial variability in DT_50_ values in a stochastic model (Leterme et al., 2007). In this work, local values of atrazine DT_50_ were randomly drawn from a distribution derived from experimental measurements in the target catchment (i.e., no spatial structure was present). The results prompted the authors to recommend taking the variability of pesticide parameters into consideration for risk assessment and pesticide regulation purposes. An obvious challenge associated with this recommendation is the current lack of relevant spatially explicit data. Some authors suggest to use sorption as a proxy for the spatial variability of pesticide degradation at the regional scale (Ghafoor et al., 2011b), but it is likely that this would be insufficient for many pesticides (see Section Controlling Factors and Co-Variants).

Several modeling frameworks for pesticide fate prediction can readily incorporate vertical heterogeneity in pesticide degradation (Ray et al., 2004; Wauchope et al., 2004). However, once again, the relevant field data to fully parameterize these models are most often not available and the simplifying assumptions made to circumvent this problem can lead to significant prediction errors (Krutz et al., 2010).

Currently, very few models thus fully incorporate realistic within-field spatial variability. One notable exception is a recent model, which includes observed three-dimensional cm-scale heterogeneity of MCPA degradation potential (Rosenbom et al., 2014). The authors conclude that, due to the generally high degradation potential in the top soil, the actual distribution of this potential does not impact MCPA leaching, unless MCPA is transported mainly in biopores with low degradation potential. This work marks an important step in the evaluation of the impact of small scale variability on pesticide fate.

### Weed control and crop yield

Considering that pesticides are typically applied to agricultural soil for weed control purposes, it is surprising that only few studies have attempted to relate spatial variability of microbial pesticide degradation to that of weed growth. Liu et al. (2002) did observe that atrazine mineralization rate was positively correlated with weed biomass and negatively with corn biomass, providing support to the notion that spatial heterogeneity in pesticide degradation can be reflected in crop yield. The presence of spatially variable degradation potential thus challenges the definition of an appropriate field-wide pesticide application dose.

Taken together, several lines of evidence thus indicate that the spatial distribution of degradation potential can modulate, at least in some conditions, the rate at which a pesticide is degraded and hence its fate and agronomic efficiency. It is then important to address the questions of how and how fast this spatial distribution can change.

## Spatial dynamics

By repeatedly sampling the same plots at different time points, researchers have proven that the spatial pattern of degraders in soil is not static but can change with time at the seasonal timescale (e.g., Hussain et al., 2013; Table S1). The spatial dynamics at this relatively long time scale is obviously affected by bacterial growth and death. However, as pointed out by Pallud et al. (2004), growth cannot by itself account for any significant bacterial spatial dynamics. Indeed, the inner soil space is so vast that colonial growth could proceed for a long time without degraders colonizing much of the soil pore space. Therefore, some other phenomena have to occur for bacteria to significantly disperse in soil. The opacity of soil and the size of the microbes make it evidently difficult to study the spatial dynamics of soil microbes *in situ*, which is why many researchers have resorted to working with experimental platforms that do not incorporate the full complexity inherent to real soil systems (e.g., Dechesne et al., 2008; Banitz et al., 2011a). It has to be noted that these studies did not always concern pesticide degraders, but the mechanisms and rates identified are likely to apply to most degrader populations as well.

Bacterial mobility is classically divided between active, termed motility, and passive, sometimes called transport. The former entails energy consumption by the bacterium (e.g., Martínez-García et al., 2014), while the latter does not.

Passive movement relies on some transporting agent. Water can transport microbes as free colloids or attached to other soil colloids (Fontes et al., 1991; Vasiliadou and Chrysikopoulos, 2011). Growing plant roots and soil fauna can also efficiently transport microbes (Gammack et al., 1992). Human action, via soil management, can also affect bacterial spatial patterns. This is exemplified by the fact that tilling was observed to homogenize the distribution of carbofuran degraders (Parkin and Shelton, 1992). Tillage not only immediately redistributes soil clods but it also provokes long-lasting changes in soil structure and porosity that may affect connectivity (Young and Ritz, 2000; Kay and Munkholm, 2011) and thus subsequently facilitate microbial dispersal.

In addition to passive transport, many bacteria can actively move, mostly thanks to dedicated cellular appendages (Jarrell and McBride, 2008). Recent years have seen significant developments in our understanding of motility in soil. Soil moisture has been confirmed as a key factor for bacterial swimming motility with motility ceasing in liquid films thinner than about 1.5 μm (Dechesne et al., 2010b). Such continuous liquid films are expected to be rather rare even in moderately wet soils. This barrier to bacterial dispersal can nevertheless be lifted by fungal hyphae, which can provide liquid films on their surface and thus effectively bridge across patches of soil devoid of hydrated pathways (Kohlmeier et al., 2005). This dispersal mechanism has been shown to greatly promote the degradation rate of PAH degraders in unsaturated soil microcosms (Wick et al., 2007). Similar accelerated bacterial degradation in the presence of fungal hyphae has recently been demonstrated for the pesticide diuron and the persistent pesticide metabolite BAM (Knudsen et al., 2013; Ellegaard-Jensen et al., 2014).

The inner soil space is considerable and its exploration by random motion is relatively slow for degrading populations with modest growth rates. Therefore, the presence of a chemotaxis system to guide degraders toward high concentrations of pesticide may significantly increase their consumption of heterogeneously distributed pesticides (Lacal et al., 2013). Many bacteria able to degrade aromatic hydrocarbons have been shown to display chemotactic behavior toward this type of pollutant (Pandey and Jain, 2002). In the case of pesticides, examples are less frequent but some well-known degradative plasmids contain elements of chemotaxis systems toward pesticides or pesticide metabolites [e.g., plasmid pJP4 carrying 2,4-D degradation pathway (Hawkins and Harwood, 2002)]. Chemotaxis toward s-triazines via a chromosomally encoded chemoreceptor has also been described (Liu and Parales, 2009). Although it is clear that some degraders have the genetic potential for chemotactic movement toward pesticides, studies showing that chemotaxis is truly efficient in soil or that it improves degradation rates are rare (Paul et al., 2006). A larger body of work exists on chemotaxis in groundwater sediments with promising results obtained in lab-scale experiments (Ford and Harvey, 2007; Singh and Olson, 2008). Sediments contrast with most soils in that hydrated water pathways are not limiting for bacterial motion. Recently, it was demonstrated that chemotaxis is possible along fungal hyphae (Furuno et al., 2010), which reinforces the “fungal highways” as a likely route for active dispersal of pollutant degraders in soil.

## Link between pesticide type, degrader community, and spatial variability: a common theme?

When comparing spatial variability across pesticides, as reviewed above, it is apparent that there is a strong link between abundance of the degrading community and its spatial distribution. High spatial variability is typically associated with populations of limited abundance, while more abundant populations are more homogeneous distributed. This is consistent with the differences existing between topsoil (hosting dispersed, abundant degrader populations) and subsoils (accommodating fewer degraders, distributed in a few patches) and with the fact that easily degradable pesticides tend to present homogeneously distributed degradation potential. Pesticide degraders are thus similar to most other types of aerobic degraders in soils, in that easily degradable compounds exhibit less patchiness in their mineralization potential than more complex ones (Dechesne et al., 2003; Hybholt et al., 2011; Monard et al., 2012). As suggested by some authors (e.g., Sørensen et al., 2003), the likely explanation for this phenomenon is that degraders of easily degradable compounds are both abundant and diverse; they thus occupy and are active in most soil microhabitats. In contrast, microbes able to degrade recalcitrant compounds are numerically rare and often poorly diverse: they are thus typically present and active in only a few favorable microhabitats. The fact that repeated pesticide applications often cause the buildup of degrader populations, resulting in larger degradation potential, suggests that, in most agricultural soils, the limiting factor for degradation is the lack of sufficiently large degrader populations, rather than inadequate environmental conditions. This point of view is, however, challenged by a study, where bacterial communities were transplanted from one soil to another and the physico-chemical properties of the soil turned out to be more influential on degradation rates than was the bacterial community (Baker et al., 2010).

## Conclusions and recommendations for future research

The spatial distribution of pesticide degraders in agricultural soils is complex, dynamic, and affected by a multitude of extrinsic (environmental conditions such as pH, organic matter content etc…) and intrinsic (e.g., microbial growth, decay) processes (Kay and Munkholm, 2011), acting at various scales. Its study and interpretation are therefore not trivial. Significant efforts have been dedicated to describe such distributions, often revealing the existence of non-random patterns. Obviously, the mere description of spatial patterns constitutes only the initial phase of research, which should then be complemented by the identification of general trends, controlling factors, by their integration into our conceptual and quantitative models of pesticide fate, and, ultimately, into our management strategies. This second, most fruitful, phase is still in its infancy. However, we will highlight and discuss here some of the most salient findings and suggest avenues for future research.

There is ample evidence that significant spatial heterogeneity in pesticide degradation potential exists within agricultural fields at scales that are compatible with tractable soil sampling. Mapping the spatial heterogeneity of degradation potential is, however, labor intensive and time consuming. Therefore, using rapidly measurable proxies that co-vary with degradation potential (such as pH for phenyl urea degraders or degradative genes (*tfd*-genes) for phenoxy acids degraders, if the sensitivity of the assay can be improved) would present advantages over the acquisition of many degradation curves, which require long incubation times. Research that aims at identifying covariates to degradation potential should thus be pursued. Emphasis should, however, also be placed on evaluating how general the identified correlations are, for example by making, and testing, predictions for plots or fields different from the ones used to establish the correlation. In addition, to make the most of these future efforts and lift the methodological barriers that currently hinder rigorous meta-analysis of the literature it would be beneficial that some key aspects of sampling protocols and data collection are standardized and that raw data obtained are shared online after publication.

The existence of a link between pesticide intrinsic biodegradability, degrader community abundance and diversity, and spatial variability in biodegradation potential seems to constitute a common theme across pesticide classes and soil types. However, this should be confirmed for pesticides that are mainly degraded co-metabolically, anaerobically, or by microbial consortia, for which literature is scarce.

Unsurprisingly, as agricultural management affects the physical and chemical aspects of bacterial microhabitat (Kay and Munkholm, 2011), within-field pesticide degradation variability is affected by many agricultural practices (pesticide application, tilling, irrigation, etc.) and can, in turn, affect the success of pest control (Liu et al., 2002). It would therefore be reasonable to consider this aspect to reach optimal pest control while minimizing pesticide application. At this point, it is however not obvious whether the benefits of applying pesticide according to the spatial pattern of degradation potential would compensate for the additional costs (notably, of mapping pesticide degradation potential).

Modeling efforts should be pursued to help us evaluate the importance of small scale heterogeneity in pesticide fate. Importantly, we need to understand under which conditions this heterogeneity needs to be taken into account and in which cases it can be ignored. This is essential to establish whether standard assessment models used for regulation of pesticide usage, which currently assume spatial homogeneity in degradation potential, need to be expanded to include spatial variability and if so, at what scale. In one recent study (Rosenbom et al., 2014), heterogeneity could safely be ignored without much underestimation of the risk of pesticide leaching to groundwater. But this was a case where pesticide degradation potential was both relatively high and relatively homogenous, and thus not representative of the range of spatial heterogeneity reported in the literature across pesticides and sites (Table 1 and Table S1). More complex models should also be developed that integrate the spatial dynamics of degraders, for example as a consequence of pesticide application. However, these spatial models cannot be easily experimentally validated as the spatial distribution of microbes is challenging to control or monitor.

The study of the spatial variability of pesticide degraders and its implications thus still present a number of technical and scientific challenges that require, maybe more than ever, a continued inter-disciplinary dialogue between soil sciences, agronomy, and microbial ecology.

## Author and contributors

Searched and analyzed literature: Arnaud Dechesne; Nora Badawi, Discussed findings: Arnaud Dechesne, Nora Badawi, Jens Aamand, Barth F. Smets; Wrote the paper: Arnaud Dechesne with contribution from Nora Badawi; Edited paper: Arnaud Dechesne, Nora Badawi, Jens Aamand, Barth F. Smets.

### Conflict of interest statement

The authors declare that the research was conducted in the absence of any commercial or financial relationships that could be construed as a potential conflict of interest.

## Supplementary material

Table S1 **Synthesis of the primary literature, including, for each reference, information on the system studied, the sampling scheme, the type of analysis, and the main observations and conclusions**.
